# Maximum Entropy Production Theorem for Transitions between Enzyme Functional States and Its Applications

**DOI:** 10.3390/e21080743

**Published:** 2019-07-29

**Authors:** Davor Juretić, Juraj Simunić, Željana Bonačić Lošić

**Affiliations:** 1 Mediterranean Institute for Life Sciences, Šetalište Ivana Meštrovića 45, 21000 Split, Croatia; 2 Ruđer Bošković Institute, Bijenička cesta 54, 10000 Zagreb, Croatia; 3 Faculty of Science, University of Split, Ruđera Boškovića 33, 21000 Split, Croatia

**Keywords:** entropy production, triosephosphate isomerase, ATP synthase, β-lactamases, bacteriorhodopsin

## Abstract

Transitions between enzyme functional states are often connected to conformational changes involving electron or proton transport and directional movements of a group of atoms. These microscopic fluxes, resulting in entropy production, are driven by non-equilibrium concentrations of substrates and products. Maximal entropy production exists for any chosen transition, but such a maximal transitional entropy production (MTEP) requirement does not ensure an increase of total entropy production, nor an increase in catalytic performance. We examine when total entropy production increases, together with an increase in the performance of an enzyme or bioenergetic system. The applications of the MTEP theorem for transitions between functional states are described for the triosephosphate isomerase, ATP synthase, for β-lactamases, and for the photochemical cycle of bacteriorhodopsin. The rate-limiting steps can be easily identified as those which are the most efficient in dissipating free-energy gradients and in performing catalysis. The last step in the catalytic cycle is usually associated with the highest free-energy dissipation involving proton nanocurents. This recovery rate-limiting step can be optimized for higher efficiency by using corresponding MTEP requirements. We conclude that biological evolution, leading to increased optimal catalytic efficiency, also accelerated the thermodynamic evolution, the synergistic relationship we named the evolution-coupling hypothesis.

## 1. Introduction

There are different formulations of the maximum entropy production principle (MEP) [1,2,3,4,5,6,7,8]. Applications to biochemical systems leading to some optimal parameters are still rare [9,10,11,12,13]. The apparent contradiction between minimum entropy production theorem (MinEP) [14] and MEP has been examined on a number of occasions [15,16]. MinEP is a very special case of the static head steady state near thermodynamic equilibrium when the linear force–flux relationship is a good approximation, net output current vanishes, and the efficiency of free-energy transduction also vanishes [17]. Free energy is stored but it cannot be used to fuel the synthesis of biological macromolecules, work of biological molecular motors, movement, and growth. The MEP principle has much more general validity both near equilibrium and far from equilibrium. The relationships among proposed MEP principles and their connections to other variational principles from irreversible thermodynamics has been frequently discussed during the last 60 years [18,19,20,21].

The application of entropy production calculations to bioenergetics and to enzyme kinetics has a special attraction for researchers interested in the question of how physics can provide an insight into life’s ability to maintain its far-from equilibrium structure through interactions with the rest of the universe. Attempts to use MinEP or to question the second law of thermodynamics in the case of photosynthesis resulted in additional controversy without contributing any additional insight to answer this question [22,23]. On the other hand, living systems are notoriously complex, and at the same time fragile and robust with the paramount importance of their biological function and evolutionary changes, which subordinates structure to function. It is probably an understatement if we say that this feature of life uniqueness is underexplored by physicists. At the same time, the application of MEP in biology opens many pertinent questions. For instance, Tyler Volk and Olivier Pauluis asked if MEP can predict dissipation details within the system and which fluxes of the system are those that are most likely to be maximized [24].

In this review, we analyzed the distribution of entropy production contributions among all catalytic steps of enzyme catalytic cycles for very different enzymes, from the triosephosphate isomerase (TIM enzyme), β-lactamases, ATP synthase, to the photochemical cycle of bacteriorhodopsin. We used our extension of Terrel Hill formalism [25,26] to the light-absorbing systems [9] for the calculations, in the case of bacteriorhodopsin. Catalytic cycles of all enzymes were analyzed with and without the application of the maximum trasitional entropy production (MTEP) theorem, which is our second extension of Hill’s formalism [13]. The MTEP theorem does not apply to the total entropy production but gives the optimal value of rate constants for the corresponding maximal value of entropy production in considered transition. The steady-state is assumed in all cases and appropriately modified Kirchhoff’s laws for biochemical circuits simplified calculations.

We asked the question of how MTEP theorem can be applied to very different examples of enzyme kinetics. Firstly, through examples of cytoplasmic enzymes, we shall illustrate how thermodynamic and kinetic criteria can be unified to obtain a good agreement between theoretical predictions and experimental measurements of kinetic rate constants. We shall also show how MTEP requirements for the last step in the catalytic cycle (the recovery step) can be used to accelerate this rate-limiting step with the concomitant increases in catalytic efficiency, free-energy transduction efficiency, output flux, and overall entropy production. Accordingly, for far-from-equilibrium systems, the relationship between entropy production and efficiency can be such that the increase of former drives the increase of the latter quantity as well. Although the kinetic and thermodynamic relationships we use are quite general, the dissection of all contributions to steady-state entropy production does point toward certain directed nano-currents as being the most important for each particular example of enzymatic free-energy conversion. The optimization of those rate-limiting steps through the MTEP requirement leads to an increase of catalytic efficiency for several enzymes that biochemists described as “perfect enzymes”. When the evolutionary distance is calculated from a putative common ancestor, more evolved enzymes show higher catalytic constants, higher catalytic efficiency, and higher overall entropy production. Bioinformatic analysis is essential for finding this connection between biological and thermodynamic evolution, which holds independently of MTEP optimization or any other entropy production principle or theorem. Secondly, for bacteriorhodopsin embedded in the topologically closed membrane, we examined how the input free energy of absorbed photons can be converted with an optimal efficiency into the electrochemical proton gradient. Power transfer efficiency can reach 90% for large enough secondary force when observed or inferred rate constants are used as input parameters. The MTEP application to the recovery step leads to an additional increase in the output flux and overall entropy production, but such an optimization procedure works better when chosen steady state is compatible with natural bacterial membrane. Similarly, the recovery step of the ATPase catalytic cycle, when ATP has been released from that membrane-bound rotary nanomotor, is also responsible for the largest contribution to total entropy production. All relevant parameters (output ATP flux, the efficiency of free-energy transduction, entropy production due to ATP synthesis) decrease for less than the optimal angular position of the transition state catalytic dwell, while an optimal angular position, as calculated from the MTEP requirement, agrees with the estimate from experiments. Taken together, these in silico results belong to quantitative conformations for the evolution-coupling hypothesis, which postulates that thermodynamic evolution accelerates biological evolution and vice versa [27].

We have listed below in Table 1 examples of MTEP optimizations considered in this study. In all of the presented examples, we took into account that enzymes cannot break time-reversal symmetry. For each transition between enzyme functional states in the forward direction, we assumed that the enzyme is capable to catalyze the back reaction as well. This dynamic equilibrium does not imply the absence of dissipation for the considered transition.

## 2. MTEP Theorem for Transitions between Enzyme Functional States

In this section, we shall repeat the derivation of the MTEP theorem for transitions between enzyme functional states as obtained by ŽBL and DJ [28]. We consider the enzyme in a steady state that can exist in any finite number of discrete functional states and can cycle among these states by going through several transitions. Generally, the affinity and the flux for a given transition can be expressed in terms of known or assumed forward and backward rate constants $k_{ij}$ and $k_{ji}$ as

(1) $$X_{ij}=k_{B}Tln\frac{k_{ij}p_{i}}{k_{ji}p_{j}}\;\left[J\right]$$

(2) $$J_{ij}/n=\left(k_{ij}p_{i}-k_{ji}p_{j}\right)\;\left[s^{-1}\right],$$

where $k_{B}$ is the Boltzmann constant, T is temperature, n is number of moles, and $X_{ij}$ is affinity felt between the species *i* and *j* of a single macromolecule, while $J_{ij}$ is the net flux for the *i*-*j* transition. The number of moles n will be taken to be one in the following text. Stationary probabilities $p_{i}$ and $p_{j}$ of the *i*-th and *j*-th state, respectively, are the fraction of macromolecular units in states *i* and *j*. Within the Hill’s diagram method [25,26] we associate each transition between two states with entropy production, which can be calculated starting from the flux and the corresponding affinity as [14]:

(3) $$\sigma_{ij}T=X_{ij}J_{ij}\;\left[Jmol^{-1}s^{-1}\right]$$

Expression (3) allows either a linear or non-linear relation between affinity and flux [21,25]. The linear relation between affinity and flux is valid for systems very close to equilibrium. However, for far from equilibrium biochemical processes, the non-linear relations between the affinities and fluxes have to be used.

The stationary probability $p_{i}$ of the *i*-th state that can be expressed through the directional diagrams $a_{ji}k_{ji}$, including the rate constant $k_{ji}$, and diagrams $b_{ij}$, excluding the rate constant $k_{ji}$. Accordingly, one may write

(4) $$X_{ij}=k_{B}Tln\frac{K_{ij}b_{ij}+k_{ij}\;a_{ij}}{b_{ji}+k_{ij}\;a_{ij}}$$

(5) $$J_{ij}=\frac{b_{ij}-b_{ji}/K_{ij}}{\Sigma /k_{ij}}$$

and obtain the entropy production in a given transition in terms of the forward kinetic constant in that transition as

(6) $$\sigma_{ij}T=k_{B}T\frac{b_{ij}-b_{ji}/K_{ij}}{\Sigma /k_{ij}}\ln\frac{K_{ij}b_{ij}+k_{ij}a_{ij}}{b_{ji}+k_{ij}a_{ij}}$$

**Figure 1:**
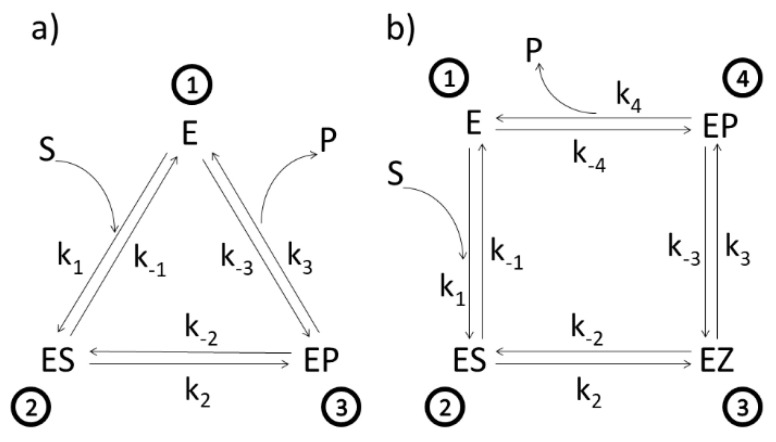
(**a**). Three-state scheme for the catalysis of β-lactamases. E, ES, EP, S, and P denote, respectively, enzyme, enzyme–substrate complex, enzyme–product complex, substrate, and product. Assumed manner of enzyme cycling is predominantly counterclockwise with forward kinetic constants k_1_, k_2_, and k_3_, and reverse kinetic constants k_−1_, k_−2_, and k_−3_. Constants k_1_ and k_−3_ are products of second-order kinetic constants and the concentrations of substrate and product, respectively. (**b**) Four-state scheme for the catalysis of triosephosphate isomerase. Additional enzyme complex is the transition state intermediate EZ.

Here, $\Sigma =k_{ij}\Sigma_{a}+\Sigma_{b}$ is the sum of the directional diagrams of all states where $k_{ij}\Sigma_{a}$ and $\Sigma_{b}$ are the sums of all state directional diagrams with or without line describing the *ij* transition, respectively. Note that each entropy production density $\sigma_{ij}$ for considered *i*-*j* transition (transitional entropy production) is a function of all the rate constants in an arbitrary kinetic process. In entropy production (6) and sum Σ, we expressed the backward constant as $k_{ji}=k_{ij}$/$K_{ij}$, where *K_ij_* is the equilibrium constant for the *ij* transition. For a given cycle between functional states, the product of all equilibrium constants defines the overall equilibrium constant *K*, and thus the overall thermodynamic force (or affinity) $X=k_{B}TlnK$. Examples of diagrams are shown in Figure 1, which will be discussed in the next section.

Results from previous research [28] show that there is a unique maximum for the entropy production of any given transition with respect to variation in the corresponding forward rate constant when we restrict our modeling to fixed values of equilibrium constants for all transitions and assume known values of kinetic constants in all other transitions. This is because the associated transition flux and affinity are, respectively, monotonically increasing and decreasing functions of the forward rate constant $k_{ij}$. In other words, there is a simple trade-off between thermodynamic flux and force. Since the equilibrium constant for the *ij* transition is constrained to the fixed value, the backward rate constant is calculated from the forward rate constant through expression $k_{ji}=k_{ij}$/$K_{ij}$ after the optimization procedure. With constant *K_ij_*, only coordinated change is possible in corresponding forward and backward kinetic constants. 

The condition for the extreme value of entropy production (6) in considered *ij* transition

(7) $$\frac{d\sigma_{ij}}{dk_{ij}}=0$$

gives the equation for the optimal value of the forward kinetic constant $k_{ij}$

(8) $$ln\frac{K_{ij}b_{ij}+k_{ij}a_{ij}}{b_{ji}+k_{ij}a_{ij}}=\frac{a_{ij}\left(K_{ij}b_{ij}-b_{ji}\right)k_{ij}\left(k_{ij}\Sigma_{a}+\Sigma_{b}\right)}{\Sigma_{b}\left(k_{ij}a_{ij}+K_{ij}b_{ij}\right)\left(k_{ij}a_{ij}+b_{ji}\right)}$$

The extreme value of the entropy production in the *ij* transition for the optimal value of the forward rate constant denoted $k_{ij}^{0}$, is then

(9) $$\sigma_{ij}\left(k_{ij}^{0}\right)=\frac{k_{B}}{K_{ij}}\frac{a_{ij}{\left(k_{ij}^{0}\right)}^{2}{\left(K_{ij}b_{ij}-b_{ji}\right)}^{2}}{\Sigma_{b}\left(k_{ij}^{0}a_{ij}+K_{ij}b_{ij}\right)\left(k_{ij}^{0}a_{ij}+b_{ji}\right)}$$

This is the maximal value of the entropy production (6) as its second derivative is negative for the optimal value of the forward kinetic constant. The above result for entropy production maximum in a given transition is valid for kinetic schemes containing multiple cycle diagrams also when we impose the same condition of fixed equilibrium constants *K_ij_* and known values of kinetic constants in all other transitions. 

Entropy production for a chosen transition is just the component of total entropy production, which is associated with a conformational transition between two functionally important enzyme states. In addition to the directional movement of enzyme atoms and amino acid residues, a conformational transition can include substrates, products, and other small molecules, atoms, or elementary particles entering or exiting the reaction pathway. In our formalism, this is taken into account in an implicit manner through second-order rate constants. We must note that maximal transitional entropy production *σ_ij_* for the transition between enzyme functional states *i* and *j* does not ensure that maximal total entropy production has been reached for a chosen steady-state system at a constant temperature.

## 3. Transitional Entropy Productions, Rate-Limiting Steps, and the Evolution or Optimization toward Higher Catalytic Efficiency 

When the majority of kinetic constants are known in a reversible kinetic scheme for enzyme cycling among several functionally important transition states, the MTEP theorem can be applied to search for answers for the following questions:

(a) Is MTEP requirement for a chosen transition producing corresponding optimized kinetic constants similar to their measured values?
(b) Can rate-limiting steps be identified as those leading to the highest increase of overall entropy production during enzyme cycling?
(c) Are rate-limiting steps connected to proton nano currents and to the shuttling of protons among enzyme, water molecules, substrate, and product?
(d) Can MTEP optimization for rate-limiting steps lead to a significant increase over the already enormous catalytic power of enzymes [29], allowing us to find a natural upper limit for the construction of perfect enzymes, for instance, by focusing on transition state mutations which can lower the activation energy for those critical steps?
(e) Can MTEP optimization for the most important free-energy conversion steps lead to high optimal efficiency for free energy storage and for free energy transduction from primary (driving) into secondary (driven) force?

Different enzymes and kinetic schemes have been considered through the years to examine these questions (Table 1). The steady-state kinetic and thermodynamic formalism, developed by Terrel Hill [25] to study free energy transduction in biology, has been recently applied to calculate entropy productions associated with all transitions between enzyme functional states in two particularly simple cases: β-lactamases [13] and triosephosphate isomerase [11]. Corresponding 3-state (Figure 1a) and 4-state kinetic schemes (Figure 1b) are just simple single-cycle schemes with only one flux. There is no primary (driving) and secondary (driven) flux-force coupling and free energy transduction efficiency cannot be defined, but we can still apply the MTEP optimization to find how enzyme catalytic efficiency can be increased. More complex kinetic schemes, including coupled cycles and fluxes, will be examined in the subsequent sections.

### 3.1. Beta-Lactamases

Let us start from the simplest and move towards more complex kinetic schemes for free-energy transducing enzymes. We used the well-known Michaelis–Menten kinetic scheme as a reversible 3-state scheme for the catalysis of β-lactamases (Figure 1a). Following Terrel Hill [25], we treated all transitions as first-order processes; forward k_i_ and reverse rate constants k_−I_ are all expressed in inverse seconds. Binding rate constant k_1_ is proportional to substrate S activity, and reverse desorption rate constant (k_−3_ for lactamases and k_−4_ for TIM) is proportional to product P activity. The ES⇄EP transition in Figure 1a is modelled as a single process. However, this catalytic process may be more complex, involving several short-living substates. When neighboring states are in rapid equilibrium with each other, the effective number of states can be reduced in the diagram. The ES⇄EP transition is then described with effective forward and backward rate constants, leading to the triangle kinetic scheme for lactamases. 

**Figure 2:**
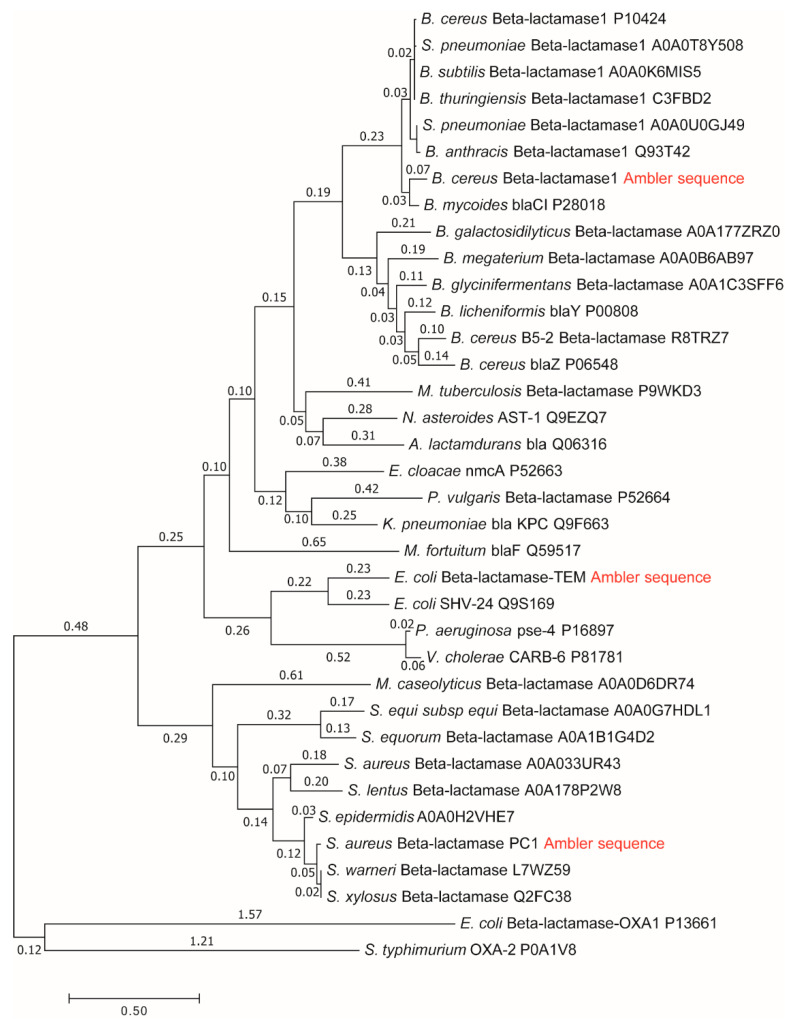
Molecular phylogenetic tree and calculation of evolutionary distances by maximum likelihood method for β-lactamases PC1, RTEM, and Lac1 [13] after using the corrected sequence of Lac1 [38]. Summing all relevant branch lengths (number above each branch) gives the following results in evolutionary distances: 1.19 for the PC1, 1.44 for the RTEM, and 1.60 for the Lac1.

**Figure 3:**
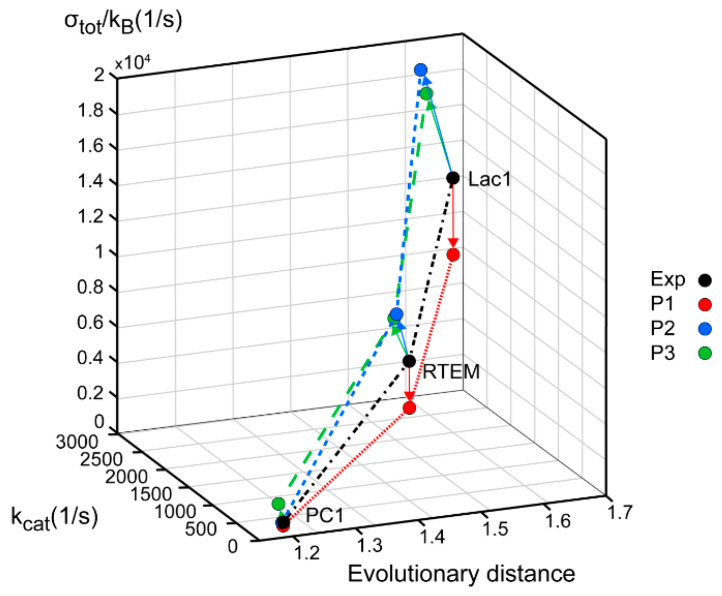
β-lactamases PC1, RTEM, and Lac1 are ranked according to their evolutionary distance from the common ancestor, catalytic constant k_cat_, and overall entropy production σ_tot_/k_B._ The corrected sequence of Lac1 [38] led to different values, but the same ranking order as we calculated previously [13]. Black circles represent values obtained from experiments and kinetic modeling, while colored circles represent the application of MTEP theorem to (**a**) the enzyme–substrate complex formation (red—P1 symbols), (**b**) the first proton transfer step leading to the formation of enzyme–product complex (blue—P2 symbols), (**c**) the second proton transfer step leading to the formation of free enzyme, and product (green—P3 symbols).

Among cytoplasmic enzymes, described by biochemists as belonging in the class of almost perfect catalysts, bacterial β-lactamases present a unique challenge to the medical community. The fast evolution of resistance mechanisms to β-lactam antibiotics is a worldwide clinical problem, but it also opens the question of why and how already “perfect” enzymes can evolve further. There are several quantitative parameters which can serve to measure evolutionary gains. Our approach to this question was to compare gains in enzyme catalytic constant $k_{\mathrm{cat}}$ and specificity constant $\frac{k_{\mathrm{cat}}}{K_{M}}$ to evolutionary distance from a putative common ancestor of all β-lactamases. In a paper about this topic [13], we used Uniprot β-lactamases sequences to construct a phylogenetic tree and to calculate their evolutionary distances. The huge and extremely useful Uniprot database of all known and inferred protein sequences did not yet exist in 1980 when Richard P. Ambler published the sequences for three β-lactamases [30]: PC1, RTEM, and Lac1. Henriette Christensen and coauthors referred to these three lactamases in their seminal 1990 paper about the determination of all the rate constants in a reversible Michaelis–Menten 3-state kinetic scheme [31]. For the first two lactamases, it was easy to find corresponding Uniprot sequences. Mature sequences (without signal peptide) differed in only one amino acid residue from Ambler’s PC1 and RTEM primary structures for the first entry into Swiss-Prot from 1988 in the case of P00807 and P62593 (coded as P00810 before 2004) sequences, and these two sequences did not change up to 2019, according to our check with the Needle algorithm (https://www.ebi.ac.uk/Tools/psa/emboss_needle/). After looking over relevant publications [32,33,34,35,36] and Ambler’s reservations [30], with respect to the identity of amino acid residues in question, we decided to use the above mentioned Uniprot sequences for further analysis. The situation is, however, different for the β-lactamase I sequence from *Bacillus cereus* 569/H. At the time of writing the 1980 paper, Richard Ambler had only the partial sequence of Lac1 as determined by David Thacher in 1975 [37]. Filling gaps was done according to unspecified personal communication, the procedure which introduced some errors in his published Lac1 sequence. These errors were corrected by M. J. Madonna, Y. F. Zhu, and J. O. Lampen, and the correct sequence was published in 1987 by Hussain et al. [38]. The corrected sequence had the highest similarity (97%) to the Uniprot P00809 entry for translated BlaY gene, the Beta-lactamase 1 (EC:3.5.2.6). This entry also did not change at all from Swiss-Prot BLAC$BACCE in 1988, an important consideration for structure–activity study based on Christensen et al. kinetic analysis published in 1990 [31]. The corrected Ambler’s sequence still had 11 sequence differences with respect to the P00809 sequence and is likely to have different evolutionary distance from a putative common ancestor for all class A β-lactamases. This is why in this work, we repeated the construction of the phylogenetic tree and recalculated the evolutionary distances for β-lactamases PC1, RTEM, and Lac1 [13] by using the corrected and published sequence of Lac1 [38]. Also, the marriage of bioinformatics, enzyme kinetics, and irreversible thermodynamics, when considering corresponding data for A class β-lactamases, had to be done by giving equal attention to each of involved sciences, including possible errors and omissions in the Uniprot database. While the science of bioinformatics was partially initiated with Prof. Amos Bairoch achievement, when he created the Swiss-Prot database in 1986 as the predecessor of the Uniprot, he realized from the very start of his Ph.D. work that databases contain errors and extracted the correct information from scientific papers (https://web.expasy.org/prolune/pdf/prolune018_en.pdf). From presented corrected version of the phylogenetic tree (Figure 2), where the same methods were used in constructing it as described previously [13], the new evolutionary distances are calculated to be: 1.19 for the PC1, 1.44 for RTEM, and 1.60 for Lac1. With these new values for evolutionary distances, we calculated for this work corrected relationships among evolutionary distances, catalytic constant, and overall entropy production (Figure 3).

The small difference observed in the evolutionary distance between our previous paper and this one can be explained as a consequence of changes in the protein sequence alignment produced by the replacement of a Lac1 sequence in these two papers. These small differences in alignment can be translated in the final distances not having the exact same value because the phylogenetic tree construction and evolutionary distance calculation are based on the sequence alignment. However, the originally calculated order of PC1, RTEM, and Lac1 evolutionary distances from the common ancestor did not change, supporting our conclusion about the connection between evolution and production of entropy.

**Figure 4:**
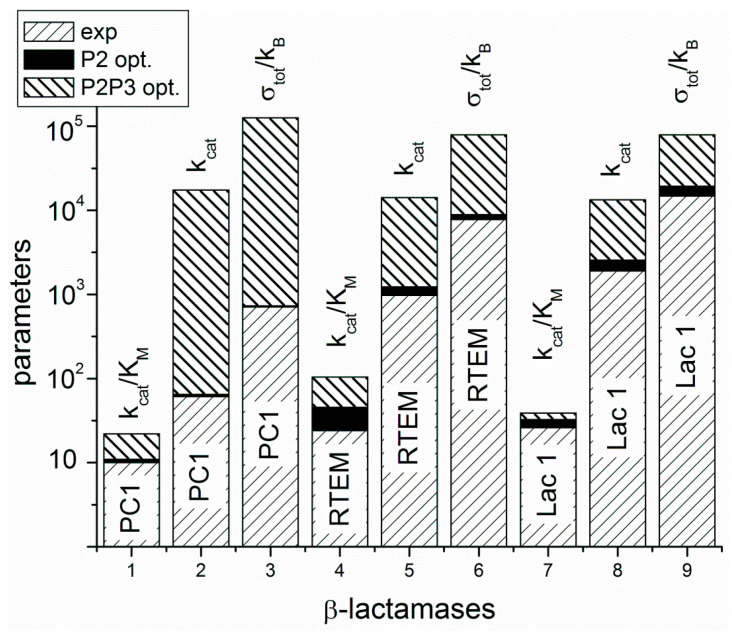
The A-class β-lactamases are located from left to right at the x-axis according to their evolutionary distance from a common ancestor, but without calculating that distance. The y-axis is used for overall performance parameters k_cat_, and k_cat_/K_M_, including overall entropy production σ_tot_/k_B_. The values obtained from a combination of experimental measurements and kinetic modeling have right-leaning stripe pattern. Optimal values obtained after maximal entropy production requirement for the acylation ES⇄EP transition are always a bit higher, but close to experimental values. That increase is represented as a black field above the experimental values. Optimal values obtained after simultaneous maximization of entropy productions for both proton transfer steps (acylation and deacylation) are represented above black fields as stacked histograms with left-leaning stripes.

Lac1 has the largest distance from the common ancestor and PC1 has the smallest distance. We can conclude, as in our recent publication [13], that the enzyme catalytic constant k_cat_ and the specificity constant k_cat_/K_M_ (regarded together as catalytic efficiency) have increased with the increase of the overall entropy production σ_tot_/k_B_ during the evolution of β-lactamases. Starting from the least evolved PC1, and going to the most evolved Lac1, the increase of kinetic and thermodynamic parameters for enzyme performance is summarized in Figure 3 and Figure 4. It is seen that optimal values obtained after maximal entropy production requirement for the acylation ES⇄EP transition are always a bit higher than the experimental values (Figure 3, P2 symbols). However, the corresponding optimal values obtained after the simultaneous maximization of entropy productions for both proton transfer steps (acylation and deacylation) are significantly higher than the experimental values (Figure 4). Thus, the optimal total entropy production and maximal entropy production for the chosen transitions between enzyme functional states are good indicators for evolutionary gains for these enzymes.

This is not the case with Shannon’s information entropy for the state probabilities p_*i*_:

(10) $$S=-\overset{3}{\underset{i}{\sum }}p_{i}lnp_{i}$$

when maximal *S* can be found (Tables 3 and 5 for PC1 and β-lactamase 1, respectively, in [13]), it has a high value which reflects almost equal probability for all three functional states. It also increased almost to the maximal value for PC1 and Lac1 after P_2_–P_3_ iterative optimization. However, in the case of the RTEM lactamase, the information entropy decreased after P_2_–P_3_ optimization.

### 3.2. Triosephosphate Isomerase

Experimental observations and measurements pointed toward desirability to include the intermediate EZ state for the triosephosphate isomerase (TIM enzyme) (Figure 1b). We used a reversible 4-state scheme, which is a straightforward generalization of the 3-state reversible scheme for β-lactamases. The TIM enzyme catalyzes the isomerization of dihydroxyacetone phosphate (DHAP) to D-glyceraldehyde 3-phosphate (GAP), an essential process in the glycolytic pathway, because only GAP physiological substrate can be subsequently used for glycolysis-derived ATP synthesis. We compared the prediction of optimal kinetic constants, catalytic constant k_cat_, specificity constant k_cat_/K_M_, and total EP applying MTEP for all four transitions between functional states, with the experimental observations for all these transitions [11,39]. The maximal entropy production (MTEP) optimization for any of the first three transitions between TIM functional states leads to decreased total entropy production. Only the MTEP optimization for the last, the product (R-glyceraldehyde-3-phosphate) release step, increases enzyme activity, specificity constant k_cat_/K_M_, and overall entropy production in comparison with experimental values (Table 2 and Table 3 of Bonačić Lošić et al. 2017 [11]). The product release step is associated with proton transport. The rate-limiting proton-transfer step is identified in this case also as the one responsible for the largest contribution to the overall entropy production during enzyme cycling. Triosephosphate isomerase is often described as a fully evolved enzyme with near-maximal reaction rate [39], but our results suggest that there is still room for additional improvement of the TIM enzyme because a reaction rate increase and the increase of the enzyme catalytic efficiency is still possible. In addition, MTEP optimizations have the potential to focus our attention at critical transitions coupled to directed movements of elementary particles, atoms, and amino acid residues, a helpful procedure to get a deeper insight into balancing flexibility and stiffness during enzyme catalysis [40].

## 4. MTEP Theorem Optimization of Transition State Parameters for ATPase

**Figure 5:**
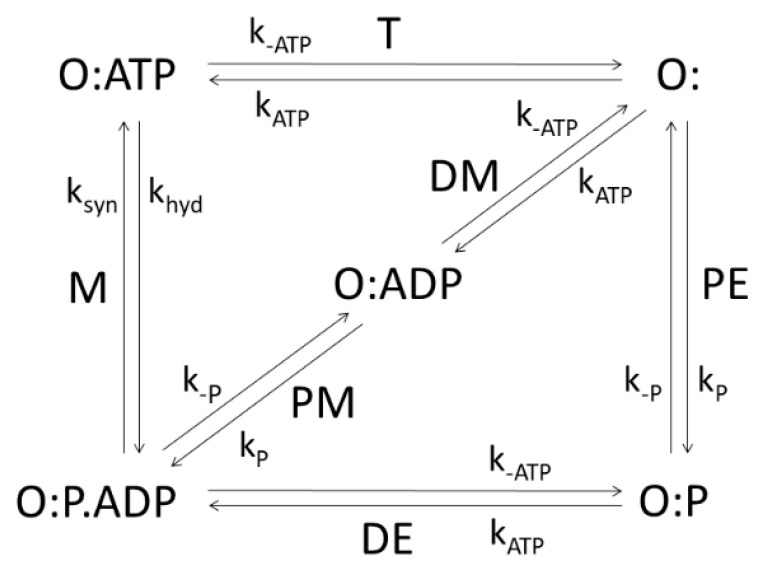
Five-state scheme for the kinetic model of ATP-ase. PE, DE, PM, DM, M, and T denote transitions between enzyme open states which may be empty (O:) or binding inorganic phosphate P, ADP, ATP. Transition M corresponds to ATP synthesis and hydrolysis.

The integral membrane protein F_0_F_1_-ATP synthase (ATPase), found universally in chloroplasts, bacteria, and mitochondria, couples transmembrane proton translocation to ATP synthesis/hydrolysis. ATPase is composed of two rotary motors: electrical F_0_ and chemical F_1_ [41]. They are coupled by elastic power transmission resulting in high efficiency of ATP synthesis. The kinetic reaction scheme for the F_0_ motor takes into account the torsional angle and elastic energy [42]. This kinetic model of ATPase [10], shown in Figure 5, consists of five functional states: empty (O:) or binding either ATP (O:ATP), ADP (O:ADP), P_i_ (O:P), or P_i_ and ADP together (O:P.ADP). The net flux $J_{ij}$ (given by expression (2)) of the transition from O:P.ADP to O:ATP equals the net rate of ATP synthesis (number of ATP molecules produced per enzyme per second), with associated forward and backward kinetic constants k_syn_ and k_hyd_, respectively. It is the M transition from Figure 5 for which the application of the MTEP theorem is particularly interesting, since it leads to free energy conversion from proton gradient into ATP synthesis. The stationary probability of the *i*-th state) (i = O:, O:ATP, O:ADP, O:P, O:P.ADP), $p_{i},$ can be obtained using Hill’s diagram method [25]. From MTEP theorem application to transition M, obtained optimal $p_{i}$ values for all transitions are listed in Table 2 together with values of entropy production and flux in each transition. States O:ATP and O:ADP have the largest stationary probabilities. As is seen from Table 2, the greatest contribution to the total entropy production comes from the recovery step, the T transition (from O:ATP to O:), when ATP is released. This transition, together with the M transition, has the highest flux equal to the net ATP synthesis flux in a steady state. We note that the optimal kinetic constant k_syn_ found from the MTEP application coincides with the corresponding one found by information entropy maximization. Furthermore, MTEP and maximum entropy (MaxEnt) modeling predict high optimal efficiency for the percentage of free-energy storage: E_out_/E_in_ = 69%. MaxEnt and MTEP optimizations for transition state parameters are in agreement with an empirical estimate about optimal angular position of about 72^o^ for the ATP-binding transition [10,42]. Therefore, joint MaxEnt and MTEP optimization results are consistent with the observed design of spinach chloroplast ATP-ase and predict an optimal working regime for this nanomotor near the inflection point of a force–flux relationship when current J is maximally sensitive to changes in the protonmotive force Δ*µ*_H^+^_.

## 5. Light-Activated Creation of the Protonmotive Force, Dissipation, and Free Energy Transduction Efficiency. The Example of Bacteriorhodopsin

**Figure 6:**
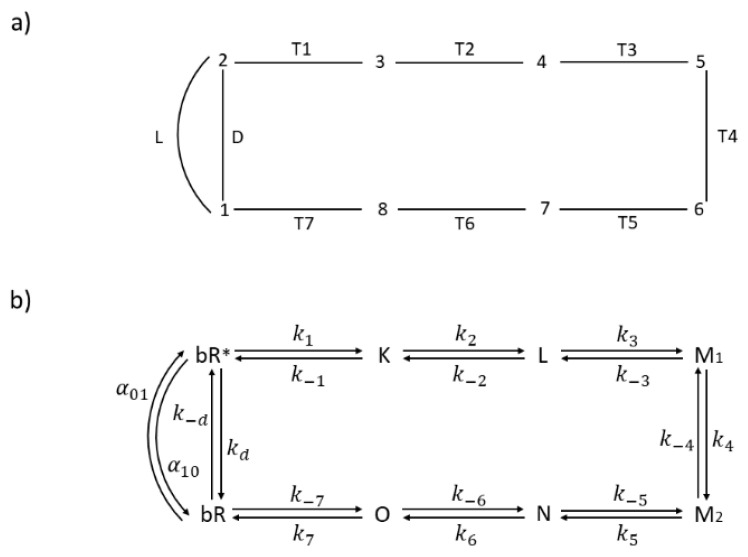
The 8-state kinetic model for the bacteriorhodopsin photocycle with (**a**) capital letters for transitions and numbers for states, and (**b**) capital letters for spectroscopic states and small letters for corresponding rate constants. Assumed manner of enzyme cycling is clockwise.

For us, absorption of a photon can trigger an eye–brain communication we call vision. For certain bacteria, photon triggers a protein quake, charge separation, electric field builds up, and photosynthesis. Although separated by a billion years of evolutionary gap, the same protein type and the same chromophore are responsible for both outcomes. It is an integral membrane protein with seven membrane-spanning helices, one of them covalently connected to the retinal chromophore. When acting as a photon detector in human rod cells, the protein is named rhodopsin. When performing the first step of photosynthesis for *Halobacterium salinarium*, the protein is named bacteriorhodopsin. Bacteriorhodopsin (bR) is the simplest solution nature found for a light-activated proton pump, which can easily perform proton active transport against the electrochemical proton gradient [43]. In its natural membrane environment, bacteriorhodopsin dissipates more than 85% of photon free energy. One can ask, why such a low efficiency of light power conversion to proton-motive power? We have seen that converting proton-motive power into ATP synthesis by an ATP-synthase is a more efficient process. Higher efficiency can be easily achieved if the stronger electric field is created, that is, greater than a minimal field of about 130 mV needed to put into rotation the ATP-synthase rotary motor for producing ATP. Photon free energy for photons with a wavelength of about 570 nm, which bacteriorhodopsin prefers to absorb, is quite high and more than enough to create a much stronger electric field. The problem with too strong an electric field is that it will cause a dielectric breakdown of a plasma membrane and cellular death. *H. salinarium* can develop a maximal electric field of about 280 mV [44]. Still, assuming that bacteriorhodopsins can be incorporated in much more robust artificial membranes, we can examine in simulations the cases when weak, strong, and super-strong secondary force is developed corresponding to the membrane potential of −195, −278, and −1185 mV, respectively. The first value of −195 mV is quite common for the membrane potential of bacteria, archaea, and mitochondria, and it is identical to one we used in earlier simulations [9]. The second value of −278 mV was also used by us earlier [45] as similar to measured maximal value for membrane potential established by *H. salinarium*, while the third and highest value of membrane potential, equal to −1.185 V, corresponds to maximal efficiency of free energy conversion, which is slightly higher than 70%. Corresponding Table 3 values are expressed in kJ/mol as respectively −18.84, −26.86, and −123 kJ/mol. We used estimated kinetic and thermodynamic parameters data for bR [46] and performed the simulations designed to answer several questions:

- Which transition step, out of seven Ti steps (Figure 6a), is associated with the greatest entropy production?
- What is the rate limiting step among all Ti transitions involved in a complex interplay of retinal, protein atoms, and water molecule movements, resulting in a proton pumping and charge separation [47]?
- When MTEP theorem is used to optimize each transition, is there a single catalytic step for which photochemical quantum yield, the efficiency of free energy conversion, and total entropy production all exhibit increased optimal values with respect to values obtained without optimization?

In references, [46] and [48] experiments and modeling were used to construct the kinetic model illustrated in Figure 6. In order to apply irreversible thermodynamics to the initial photon absorption step, we introduced the excited state bR^*^, light activated transition L from ground to excited state, and non-radiative transition D back to ground state (Figure 6b). For details, see our earlier papers [9,45] about this extension of Hill’s formalism [25] to the light-absorbing systems. Proton transfer and charge separation take place in the productive T1 to T7 pathway. In a reversible model of van Stokkum and Lozier [46], all thermodynamic and kinetic parameters (equilibrium constant, forward, and reverse kinetic constants) have been estimated for T2 to T6 transitions. They estimated the forward constant k_7_ in the last recovery transition (T7) as k_7_ = 700 s^−1^. To get Table 3 values, we used that and other estimated parameters by van Stokkum and Lozier for pH = 7, while τ = 4 ps time-constant estimate by Nango et al. [48] was used to calculate the forward constant k_1_ as k_1_ = 2.5 × 10^11^ s^−1^. With a choice of K_7_ = 2 × 10^7^ for recovery transition equilibrium constant, the remaining constants for the T1 transition could be easily calculated from the requirement that the product of all equilibrium constants in the charge separation cycle must be equal to exp(X_sec_/k_B_T) [25]. We have chosen the kinetic constant k_d_ for non-radiative D transition as k_d_ = 10^8^ s^−1^ and the light-absorption rate α_01_ = 100 s^−1^ by following our 2003 choice [9] for modeling bacteriorhodopsin photocycle with system being at room temperature T = 298.16 K. Equilibrium constants in the light cycle L–D are found as K_L_ = exp(hv/k_B_T_R_) and K_D_ = exp(hν/k_B_T), where T_R_ is an effective temperature, which is higher than T and increases with increased light absorption rate α_01_ and increased light intensity I = J_L_ [9].

According to van Stokkum and Lozier [46], when T1 transition is not considered, most free energy is dissipated in the recovery step. This is confirmed by our calculations of entropy production for each step in the charge separation pathway when picoseconds relaxation from excited state bR^*^ to K_590_ spectroscopic state and associated entropy production σ_1_ is not considered. 

**Figure 7:**
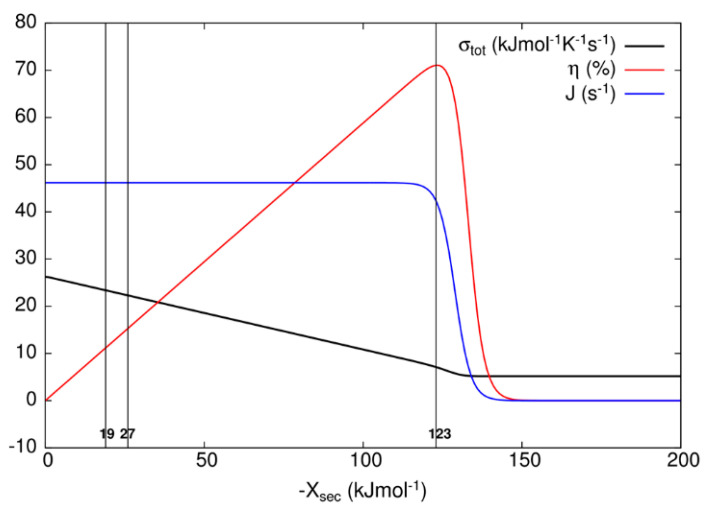
Dependence of the overall entropy production σ_tot_, efficiency η, and flux J on secondary force X_sec_ for bacteriorhodopsin photocycle. The vertical lines represent cutoffs at secondary force values of −18.84, −26.86, and −123 kJ/mol, respectively, corresponding to data presented in Table 3. Secondary force value of −123 kJ/mol corresponds to maximal efficiency η.

After examining all results, we concluded that only the MTEP application to recovery step (transition T7) for the kinetic model of the bacteriorhodopsin photocycle leads to an additional increase in the output flux and overall entropy production, with respect to values obtained without optimization (Table 3). We note that state probability p_2_ of the excited state bR^*^ is very small because the transition from state 2 to state 3 is very fast with large forward kinetic constant k_1_ and correspondingly large equilibrium constant, while thermal relaxation to the ground state bR is also fast for the D transition. In order to choose representative secondary forces for the construction of Table 3 and Figure 7 for the kinetic model of the bacteriorhodopsin photocycle, we have investigated how flux J, entropy production σ_tot_, and efficiency η vary as the secondary force X_sec_ varies. As it is seen from Figure 7, flux stays almost constant and entropy production decreases as the efficiency increases from zero to its maximal value when one varies secondary force from zero to −123 kJ/mol. As one further varies secondary force, flux and efficiency fall to zero and entropy production falls to a finite, almost constant, low value. When oriented bacteriorhodopsins are incorporated in robust bioelectronic devices, a high efficiency can be reached [49], with an additional bonus of smaller entropy production, that is, considerably less heating with a very small decrease in produced proton current. Another bR-based bioelectronic application is in the field of volumetric optical memory [50]. It is using different means to channel the same recovery transition T7, which we found to be critical for the optimization, into a branched pathway enabling writing, reading, and erasing information. From our analysis, it follows that the MTEP optimization for the T1 transition can increase the O state occupancy and efficiency of a branched recovery pathway (not shown).

## 6. Discussion

By using a classic combination of irreversible thermodynamics and enzyme kinetics [25,26], we examined the question of how thermodynamic evolution is connected to biological evolution. With our extensions of Hill’s formalism (MTEP theorem, light-absorbing systems), it is possible to identify those rate-limiting transitions that are leading to an increase in total entropy production after MTEP optimization, to check if optimized rate constants are comparable to measured rate constants, and also to see if enzyme catalytic efficiency can be increased. It is also possible to examine the structure-dynamic and structure-function connection from a fresh outlook. All examined examples have in common that enzyme structure enables the transfer of protons among several critical amino acid residues and water molecules, resulting in a directed nano-current of protons. Proton flux would not exist without imposed external force that is keeping the whole system safely away from thermodynamic equilibrium. For simpler cytoplasmic enzymes, a substrate–product pair is maintained in a homeostatic disequilibrium. Nonequilibrium substrate and product concentrations are responsible for chemical affinity as a single driving force producing the flux of product molecules. This is the case with TIM enzyme and β-lactamases. More complex enzymes are usually membrane-embedded proteins capable of converting the primary force–flux couple into a secondary force–flux couple. This is the case for ATP-ase and bacteriorhodopsin. Entropy production calculations can already lead to the identification of rate-limiting steps among all transitions without performing any optimization if a complete set of rate constants is known, but MTEP theorem helps for frequent cases when some rate constant is not known. The last catalytic step, that is, the recovery of the enzyme to its original ground state, is often at the same time the rate-limiting step and the cause for the highest dissipation among all other transitions. 

Repeated calculations for evolutionary distances of three lactamases have shown the robustness of previously found proportionality among distances from a common ancestor, catalytic constant, catalytic efficiency, and total entropy production [13]. This result follows from a straightforward application of Hill’s formalism for entropy production calculations [25] in combination with a bioinformatic analysis without recourse to any optimization technique. The acylation and deacylation steps with concomitant proton shuttles are the rate limiting for β-lactamases PC1, RTEM, and Lac1 and the most important contributors to overall entropy production. When these two steps are optimized by using the MTEP theorem, the catalytic activity (the turnover number) can be increased from one to two orders of magnitude (Figure 4). In practice, this can be achieved by finding mutations which can lower the activation energies for proton shuttles, that is, by finding the specific transition state mutants for accelerating the acylation and deacylation catalytic steps. It is not something we want to do of course, because the evolvability of β-lactamases has already led to the worldwide spread of multidrug-resistant bacterial pathogens. This happened despite common opinion among biochemists that wild-type lactamases are nearly perfect enzymes. However, “super-lactamases” predicted by the application of MTEP theorem are uncomfortably close to or even inside the forbidden diffusion-limit region. Still, this simple application of irreversible thermodynamics to enzyme kinetics led us directly to the main driving engine for enzyme evolution: the transition-state mutations for rate-limiting steps. In the examples of β-lactamases and TIM enzyme entropy production, calculations can identify those crucial catalytic steps, which are at the same time the most efficient in performing catalysis and in dissipating free-energy gradients. 

For ATP-ase kinetic scheme (Figure 5), we have achieved the best agreement between MTEP and MaxENT predictions and experimental findings with the caveat (DJ personal communication with Oliver Pänke and Bernd Rumberg) that empirical estimate for the relative angular position of the catalytic dwell κ (corresponding to angular position 72^o^) was not very accurate. Also, in the ATP-ase kinetic scheme, the recovery step when ATP molecule is released from the F_1_ rotor is accompanied with the highest contribution to overall entropy production (Table 2). ATP-as nanomotors are biologically very old inventions. Both respiration and initial photosynthetic steps converge toward creating protonmotive force, which can be used by ATP-ase to convert spontaneous inward-directed proton nano currents first into rotation of their stator subunits, then into elastic energy and finally into the pushing together of ADP and inorganic phosphate to create ATP molecule without the hindrance of water molecules. This principle is observed by all ATP-ase molecular motors including the ATP-ase from spinach chloroplasts studied by us [10] and ATP-ase from *Halobacterium salinarium*. The efficiency of converting protonmotive force free energy into free energy of far-from-equilibrium ADP–ATP concentrations is truly amazing. It was estimated to be close to 70% and calculated by us as the optimal value of 69% as the result of MTEP and MaxENT maximization.

When known and estimated values for kinetic constants are used to calculate the contribution to entropy production of all transitions in the 8-state bacteriorhodopsin photocycle (Table 3), the last recovery step is responsible for the highest contribution, but only when developed electrochemical proton gradient becomes too high for the photosynthetic cell to maintain the integrity of cytoplasmic membrane. It will surely experience a dielectric breakdown at a considerably lower value of the transmembrane electric field. For more realistic values of membrane potential, the contribution of the bR* to K transition T1 (Figure 6) is more important for total entropy production. In *Halobacterium salinarium*, the membrane-embedded proton pumps of bacteriorhodopsin and ATP-ase are coupled together through proton flux in the simplest photosynthetic circuit created by natural evolution. Coming back to the efficiency of power transduction by bacteriorhodopsin, we can see that in the hypothetical case of engineered membrane capable of withholding the membrane potential of −1.185 volts (corresponding to −123 kJ/mol secondary force for the maximal efficiency of 71%, see Table 3), an overall photosynthetic efficiency would be close to 50%. Naturally evolved photosynthetic organelles, cells, and organisms do not need such a high efficiency for producing ATP molecules, but our civilization has an urgent need to use renewable free energy coming from our sun in the most efficient way possible. If these proton pumps can provide an inspiration for how to achieve such a goal, the bonus will be a decreased level of entropy production, because with the higher secondary force, we are actually approaching something similar to the static head state for nonlinear force–flux relationships, as can be seen from Figure 7 and earlier calculations [15].

It is argued in the paper by Jennings et al. [51] that plant photosystem I, which performs primary charge separation in around 40 ps, does so with minimal entropy production. While the transfer of photon free energy into bond twisting (in the case of bacteriorhodopsin) or electron–hole excitation (for plant photosynthesis) is the obligatory first step (in femtoseconds) of quantum nature, which is associated with very low dissipation, the biologically most relevant step is the light-activated proton transport. Proton pumping creates the protonmotive force. The ratio of output protonmotive power to input power provided by photons is biologically much more relevant quantity than high quantum efficiency, promoted by Jennings et al. [51] as the evidence for minimal or even negative entropy production [23]. Indeed, within restrictions of our model for bacteriorhodopsin photocycle, the efficiency of free energy transduction in the biological range of membrane potentials does not exceed 16%, while the photochemical yield or quantum efficiency does not decrease below 99%. Lower than 16% efficiency is easy to realize with an increased dissipation rate (Figure 7). Similar behavior is observed by Baiesi and Maes [52], that is, efficiency decreases when external constraints are changed to move the system away from some optimal (low) dissipation level.

In bioenergetics, it is generally accepted that living cells are superior entropy producers with respect to an equivalent volume of some average star, like our sun. The dissipation is tightly coupled to accurate signaling [53], sensory adaptation [54], many regulatory feedback cycles in biochemistry, and kinetic proofreading [55,56]. One can ask the question: is life a constant struggle against the tendency to produce entropy or not? [57]. The connection between evolution and increased entropy production is not restricted to the living world. More complex structures emerge with a greater distance from equilibrium for many different open systems. Evolution of galaxies, stars, planets, life, society, and machines is connected to the slow or the fast increase in energy rate density with time [58]. It is well known that black holes are central players in the evolution of galaxies, and also the most important contributors to entropy increase in the universe [59]. Dynamic description of complex biochemical and physical systems, including oxidative phosphorylation [60], metabolic networks [61], and the earth’s global climate [62] is in accordance with maximization of entropy production.

The MTEP requirement may be useful as a simple optimization method founded in physics to study the evolutionary optimization of enzymatic reactions. We can assume that the ATP-synthase evolved in accordance with the MTEP theorem and with the statistical principle of maximum Shannon’s entropy [10]. Shannon’s information entropy of discrete enzyme states increased in accordance with increased evolutionary distance for β-lactamases. However, unlike the maximal entropy production for transitions between functional states, the maximal Shannon’s information entropy cannot always be found if we do not apply additional restrictions. As an example, MTEP optimization in the recovery T7 transition for the kinetic model of the bacteriorhodopsin photocycle did not result in increased Shannon’s information entropy. In Table 4 we have compared the calculations of Shannon’s entropy and overall entropy production for β-lactamases, TIM enzyme, ATP-ase, and bR, in the case of input parameters (kinetic constants), were estimated from experiments, and when their optimal values were obtained after MTEP optimization for the rate-limiting catalytic transition. The TIM enzyme obviously works not so far from equilibrium, but its Shannon entropy is far from the theoretical maximum for the 4-state kinetic model. The ATP-ase and bacteriorhodopsin are less amenable than β-lactamases to MTEP optimization capable of increasing kinetic constants. This may be due to billions of years which evolution has had to perfect these proton pumps. 

Several conclusions follow from found connections between entropy production and enzyme performance parameters honed by the forces of natural selection. Firstly, the dissection of entropy production contributions for evolutionary related enzymes cycling among functional states is not a direct application of the MEP principle. The MEP principle applicability is restricted to small-time intervals and volume elements [21], while gains in catalytic activity, biological complexity, and corresponding increases in entropy production density over eons [13,63] are not restrained within these bounds. The MTEP theorem application can still be helpful for considerations of how natural or human design can increase overall entropy production and at the same time, improve the steady-state operation of bionanomachines. Secondly, we can conclude that the application of the MTEP theorem in enzyme kinetics and bioenergetics produced three main insights: (a) an intimate connection exists among far-from-equilibrium-situation, nonlinear force–flux relationships, concomitant increases in entropy production, and efficiency increase of free-energy transduction, (b) entropy production calculations for each transition between functional states helps to identify the rate-limiting steps among all enzymatic transitions leading to product formation, and (c) evolutionary distance calculations support the evolution-coupling hypothesis [27] when correlated to concomitant catalytic efficiency and entropy production increase.

## Figures and Tables

**Table 1:** The list of enzymes and kinetic models to which the MTEP theorem has been applied, with a brief outcome of performed optimizations.

The Enzyme (C or M) *	States	Transitions	Reference	Outcome
β-Lactamase PC1 β-Lactamase RTEM β-Lactamase Lac1 (all C)	3	3	[13,28], this work	Evolutionary distance and overall entropy production rank these three lactamases in the order: Lac1 > RTEM > PC1. The same raking is found for optimal values of catalytic constant and for the catalytic efficiency.
Triosephosphate isomerase (C)	4	4	[11]	The product release step identified as the rate-limiting step. Its optimization led to a 30% increase in enzyme activity, specificity constant k_cat_/K_M_, and overall entropy production.
ATP synthase (M)	5	6	[10]	High optimal efficiency of free energy storage *η* = 0.69. Agreement with an empirical estimate for gearing ratio and optimal angular position for the ATP-binding transition.
Bacteriorhodopsin (M)	8	9	[9,15], this work	The MTEP application for the recovery step leads to an additional increase in the output flux, efficiency, and overall entropy production

* C = cytoplasmic protein converting substrate to product. M = integral membrane protein converting primary flux-force couple into a secondary flux-force couple.

**Table 2:** State probabilities, entropy productions, and fluxes for a kinetic model of ATP-ase, which was optimized for maximal transitional entropy production in the transition M.

State Probabilities		Transition Entropy Productions		Transition Fluxes	
p_O:_	0.04	σ_T_ (kJmol^−1^K^−1^s^−1^)	3.175	J_T_ (s^−1^)	81.06
p_O:ATP_	0.3	σ_M_ (kJmol^−1^K^−1^s^−1^)	1.117	J_M_ (s^−1^)	81.06
p_O:ADP_	0.49	σ_DM_ (kJmol^−1^K^−1^s^−1^)	0.158	J_DM_ (s^−1^)	72.45
p_O:P_	0.01	σ_PM_(kJmol^−1^K^−1^s^−1^)	0.121	J_PM_ (s^−1^)	72.45
p_O:P.ADP_	0.16	σ_PE_ (kJmol^−1^K^−1^s^−1^)	0.026	J_PE_ (s^−1^)	8.62
		σ_DE_ (kJmol^−1^K^−1^s^−1^)	0.007	J_DE_ (s^−1^)	8.62
		σ_tot_ (kJmol^−1^K^−1^s^−1^)	4.604		

**Table 3:** Kinetic models of the bacteriorhodopsin photocycle without and with MTEP optimization in the recovery T7 transition.

Parameters *	X_sec_ = −18.84 kJmol^−1^		X_sec_ = −26.86 kJmol^−1^		X_sec_ = −123 kJmol^−1^	
	No Optimization	T7 Optimization	No Optimization	T7 Optimization	No Optimization	T7 Optimization
k_7_ (s^−1^)	700	1750	700	1750	700	1670
σ_L_ (kJmol^−1^K^−1^s^−1^)	2.1	2.5	2.1	2.5	0.7	0.8
σ_D_ (kJmol^−1^K^−1^s^−1^)	9.8·10^−3^	1.2·10^−2^	9.8·10^−3^	1.2·10^−2^	0.5	0.5
σ_1_ (kJmol^−1^K^−1^s^−1^)	14.7	16.3	13.4	14.9	7.3·10^−3^	9.0·10^−3^
σ_2_ (kJmol^−1^K^−1^s^−1^)	2.5·10^−3^	2.9·10^−3^	2.5·10^−3^	2.9·10^−3^	2.3·10^−3^	2.7·10^−3^
σ_3_ (kJmol^−1^K^−1^s^−1^)	2.5·10^−2^	2.9·10^−2^	2.5·10^−2^	2.9·10^−2^	2.3·10^−2^	2.7·10^−2^
σ_4_ (kJmol^−1^K^−1^s^−1^)	4.9·10^−2^	5.7·10^−2^	4.9·10^−2^	5.7·10^−2^	4.4·10^−2^	5.2·10^−2^
σ_5_ (kJmol^−1^K^−1^s^−1^)	0.5	0.6	0.5	0.6	0.4	0.6
σ_6_ (kJmol^−1^K^−1^s^−1^)	0.4	0.7	0.4	0.7	0.3	0.6
σ_7_ (kJmol^−1^K^−1^s^−1^)	5.7	5.9	5.7	5.9	5.1	5.3
σ_tot_ (kJmol^−1^K^−1^s^−1^)	23.4	25.9	22.2	24.5	7.1	7.8
J (s^−1^)	46.2	51.1	46.2	51.1	42.1	46.5
η (%)	11.1	11.1	15.8	15.8	71.1	71.2
J/J_L_ (%)	99.96	99.96	99.96	99.96	98.08	98.28
A/A_oc_ (%)	91.95	91.95	91.95	91.95	97.33	97.20
S	1.28	1.20	1.28	1.20	1.22	1.14
p_1_	0.46	0.51	0.46	0.51	0.51	0.55
p_2_	1.9·10^−10^	2.1·10^−9^	1.9·10^−10^	2.1·10^−9^	7.8·10^−9^	2.1·10^−9^
p_3_	0.02	0.02	0.02	0.02	0.02	0.02
p_4_	0.06	0.07	0.06	0.07	0.06	0.06
p_5_	0.10	0.11	0.10	0.11	0.09	0.10
p_6_	0.13	0.14	0.13	0.14	0.12	0.13
p_7_	0.15	0.13	0.15	0.13	0.14	0.12
p_8_	0.07	0.03	0.07	0.03	0.06	0.03

* Entropy productions, fluxes, efficiencies, entropies, and state probabilities for three representative values of secondary force in 8-state kinetic models of the bacteriorhodopsin photocycle without and with MTEP optimization in the recovery T7 transition. A/A_oc_ is affinity transfer efficiency, η is free-energy transduction efficiency, and J/J_L_ is the photochemical yield (quantum efficiency) [9].

**Table 4:** Steady state Shannon entropy and entropy production for β-lactamases, TIM enzyme, ATP-ase, and bR.

		PC1	RTEM	Lac1	TIM	ATPase	bR		
X_sec_ (kJmol^−1^)							−18.84	−26.86	−123
Shannon’s entropy	exp	0.68	0.74	0.86	0.31	1.17	1.28	1.28	1.22
	Opt *	0.65	0.54	0.78	0.27	1.17	1.20	1.20	1.14
	max	1.10	1.10	1.10	1.39	1.61	2.08	2.08	2.08
σ_tot_ (kJ/(mol·K·s))	exp	5.73	56.18	120.76	0.08	4.70	23.41	22.17	7.08
	Opt *	6.08	73.67	161.11	0.12	4.60	25.92	24.54	7.75

* MTEP optimization performed only for rate-limiting transitions.
